# Antitumour Effects of Selected Pyridinium Salts on Sensitive Leukaemia HL60 Cells and Their Multidrug Resistant Topoisomerase II-Defective HL60/MX2 Counterparts

**DOI:** 10.3390/molecules27165138

**Published:** 2022-08-12

**Authors:** Jolanta Tarasiuk, Dorota Kostrzewa-Nowak, Wojciech Żwierełło

**Affiliations:** Department of Biochemistry, Faculty of Biology, University of Szczecin, 3c Felczaka St, 71-412 Szczecin, Poland

**Keywords:** pyridinium salts, human promyelocytic leukaemia HL60 cells, multidrug resistance, topoisomerase II, reactive oxygen species, apoptotic and lysosome-dependent cell death

## Abstract

Multidrug resistance (MDR), having a multifactorial nature, is one of the major clinical problems causing the failure of anticancer therapy. The aim of this study was to examine the antitumour effects of selected pyridinium salts, 1-methyl-3-nitropyridine chloride (MNP) and 3,3,6,6,10-pentamethyl-3,4,6,7-tetrahydro-[1,8(2H,5H)-dion]acridine chloride (MDION), on sensitive leukaemia HL60 cells and resistant topoisomerase II-defective HL60/MX2 cells. Cell growth was determined by the MTT test. Intracellular ROS level was measured with the aid of 2′,7′-DCF-DA. The cell cycle distribution was investigated by performing PI staining. DSB formation was examined using the γ-H2AX histone phosphorylation assay. The activity of caspase-3 and caspase-8 was measured with the use of the FLICA test. The assays for examining the lysosome membrane permeabilization were carried out with the aid of LysoTracker Green DND-26. Both studied compounds exerted very similar cytotoxic activities towards sensitive HL60 cells and their MDR counterparts. They modulated the cellular ROS level in a dose-dependent and time-dependent manner and significantly increased the percentage of sensitive HL60 and resistant HL60/MX2 cells with sub-diploid DNA (sub-G1 fraction). However, the induction of DSB formation was not a significant mechanism of action of these pyridinium salts in studied cells. Both examined compounds triggered caspase-3/caspase-8-dependent apoptosis of sensitive HL60 cells and their MDR counterparts. Additionally, the findings of the study indicate that lysosomes may also participate in the programmed death of HL60 as well as HL60/MX2 cells induced by MDION. The data obtained in this work showed that both examined pyridinium salts, MNP and MDION, are able to retain high antileukaemic effects against multidrug resistant topoisomerase II-defective HL60/MX2 cells.

## 1. Introduction

Despite the great progress in the development of antitumour drugs, there is still a genuine need to obtain new agents with higher therapeutic efficacy. Specifically, a huge effort has been made to design new potent drug candidates that can retain activity against tumours resistant to structurally various chemotherapeutics having different modes of action, since the emergence of multidrug resistance (MDR) is one of the main causes of clinical failure in the treatment of cancer patients [1,2,3]. The MDR phenomenon has a multifactorial nature associated with several molecular mechanisms, such as: (i) overexpression of membrane transporters belonging to ATP-binding cassette (ABC) protein family (e.g., P-glycoprotein, P-gp; multidrug resistance-associated protein 1, MRP1, and breast cancer resistance protein, BCRP) excluding drugs out of cells, (ii) alterations in drug target molecules (e.g., changes in level and/or activity of topoisomerase II), (iii) drug sequestration or inactivation, (iv) intensification of DNA damage repair processes, and (v) cell death inhibition (reviewed recently by Assaraf [4]).

A large part of research in the field of developing new anticancer drugs is focused on the search for pyridinium compounds of both natural [5,6,7] and synthetic [8,9,10,11,12,13] origin. There are many reports showing their efficacy against various types of haematological and solid tumours. Among them are marine Raniera sari sponge-derived polymeric alkylpyridinium salts exhibiting high activity towards lung cancer cells [5,7] and 1(10-aminodecyl) pyridinium salt from marine actinomycete exhibiting potent cytotoxic activity in vitro against various solid (e.g., cervix, breast, and brain) tumour cell lines [6]. There is also an increasing amount of experimental data demonstrating that synthetic compounds possessing various pyridine-based scaffolds have promising antitumor properties. For example, it was found that fluorinated pyridinium salt-based hydrazones were able to induce apoptosis in breast and colorectal tumour cells [8]. It has also been reported that synthetic cyanine dyes containing pyridinium salt moieties exhibit promising biological potency against a large panel of solid (e.g., liver, cervical, breast, pancreas, kidney, and lung) tumour cell lines [9]. There are also several studies demonstrating that some pyridine compounds have promising antileukaemic activity. For instance, it was found that pyridyl analogues of CDDO-imidazolide were able to induce differentiation and apoptosis of U937 leukaemia cells [10]. Another group of compounds, having a pyridine-based scaffold, namely pyrano-pyridine derivatives, exerted cytotoxic activity against K562 leukaemia cells [12]. Promising antiproliferative properties against a large panel of leukaemic (U-937, MOLT-3, MOLT-4, K-562, and NALM-6) cell lines have also been reported from some synthetic α-bromoacryloylamido indolyl pyridinyl propenones [11] and aryl pyrazole derivatives [13].

Various cellular mechanisms of action of compounds having pyridine-based functional groups are proposed. Among them, one of the most commonly suggested is the dysregulation of mitochondrial potential triggering apoptosis [14,15]. There are also several studies indicating that the activity of these agents against tumour cells is associated with the ability to shift the NAD(P)H/NAD(P)^+^ equilibrium in non-enzymatic and enzyme-mediated processes, which leads to serious disorders in cell metabolism, by affecting the rates of NAD(P)H- and NAD(P)^+^-dependent reactions [16,17,18]. It has also been demonstrated that the antitumour activity of 3-alkylpyridinium polymers from marine Raniera sari sponge is related to their capability to interfere with the cholinergic system activating proapoptotic pathways in non-small cell lung carcinoma cells [5,7]. Furthermore, it was found that the elaborated synthetic multifunctional, membrane-interactive pyridinium salt was able to disturb lipid membranes and induce the death of glioma primary cells [19]. It has been also reported that the antitumour activity of pyridinium-hydrazone derivative agents may be related to their ability to inhibit autophagic flux [20].

However, although there is an increasing body of experimental data indicating the therapeutic potential of many pyridine scaffold-based agents towards sensitive solid and haematological tumours, so far, little is known about their activity against cancers resistant to conventional drugs. In fact, there are only a few reports demonstrating their ability to retain efficacy against MDR cells. A recent study published by Yun et al. showed that the cinnamaldehyde derivative containing pyridinium moiety, CB-PIC, was able to induce apoptosis in paclitaxel-resistant lung cancer H460/PT cells, doxorubicin-resistant breast cancer MCF7/Adr, and colon cancer HCT15/cos cells by suppressing P-gp expression through inhibition of STAT3 and AKT signalling [21]. Some promising results were also obtained for the cationic ceramide analogue containing pyridinium moiety, LCL124, which showed that this agent caused mitochondrial depolarization and induced apoptosis in gemcitabine-resistant pancreatic cancer cells [15] as well as for the developed multifunctional, membrane-interactive pyridinium salt exhibiting potent antitumour activity towards glioma primary cells resistant to lomustine, vincristine (VINC), and doxorubicin (DOX) [19].

**Figure 1:**
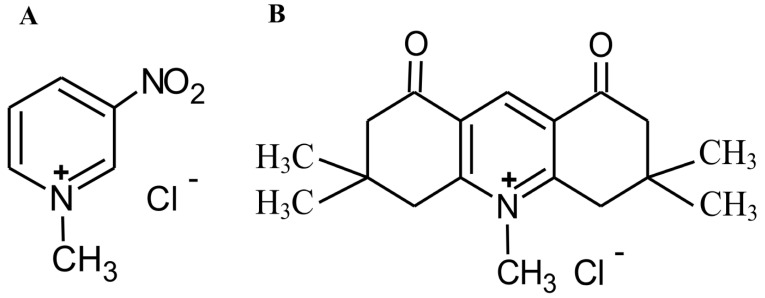
Structure of MNP (**A**) and MDION (**B**).

The selected pyridinium salts, 1-methyl-3-nitropyridine chloride (MNP) and 3,3,6,6,10-pentamethyl-3,4,6,7-tetrahydro-[1,8(*2H,5H*)-dion]acridine chloride (MDION) (Figure 1), being the subject of the present study, also have a promising potential for the treatment of neoplasm diseases. These compounds are able to disturb cellular metabolism by affecting the rates of NAD(P)H- and NAD(P)+-dependent reactions [22,23,24,25]. Their antitumour activity has been demonstrated against murine lymphocytic leukaemia L1210 cells [24] and human promyelocytic leukaemia HL60 cells [23]. Furthermore, it was found that MNP and MDION retain antileukaemic activity towards resistant sublines exhibiting the mechanisms of MDR associated with the overexpression of ABC-exporting pumps, namely, P-gp (HL60/VINC) and MRP1 (HL60/DOX) [23]. However, their activity and mechanisms of action against MDR tumour cells presenting other phenotypes of this multifactorial phenomenon have not yet been explored. Therefore, the aim of the present work was to determine the effects of these selected salts, MNP and MDION, against resistant HL60/MX2 cells possessing the mutated α isoform of topoisomerase II and lacking the β isoform of this enzyme.

## 2. Results

### 2.1. The Ability of MNP and MDION to Inhibit the Growth of Sensitive HL60 and Resistant HL60/MX2 Leukaemia Cells

In the first part of the study, the ability of MNP and MDION to inhibit the growth of human promyelocytic leukaemia sensitive HL60 cell line and its resistant subline HL60/MX2 was examined after a 72 h incubation. IC_20_, IC_50,_ and IC_90_ values (MNP and MDION concentrations required to inhibit 20%, 50%, and 90% of cell growth, respectively) and resistance factor (RF) values determined for these pyridinium salts are given in Table 1.

It was found that MNP and MDION exhibited very similar activities in inhibiting the growth of sensitive HL60 and resistant HL60/MX2 cells. The RF values determined for these compounds were close to 1.0. However, it was found that MNP was several times more active than MDION towards sensitive HL60 cells and their MDR counterparts.

### 2.2. The Effect of MNP and MDION on the ROS Level in Sensitive HL60 and Resistant HL60/MX2 Leukaemia Cells

The effect of MNP and MDION on ROS level in sensitive HL60 and resistant HL60/MX2 cells was studied using the 2′,7′-dichlorofluorescein diacetate (2′,7′-DCF-DA) as a fluorescent indicator of overall intracellular ROS. The cells were incubated with the examined pyridinium salts used at IC_20_ and IC_50_, respectively, for up to 72 h and the cellular level of ROS were determined at indicated time points (0, 2, 6, 24, 48, and 72 h), taking into account the dynamic changes in the oxidoreductive status of the cells exposed to exogenous agents.

**Figure 2:**
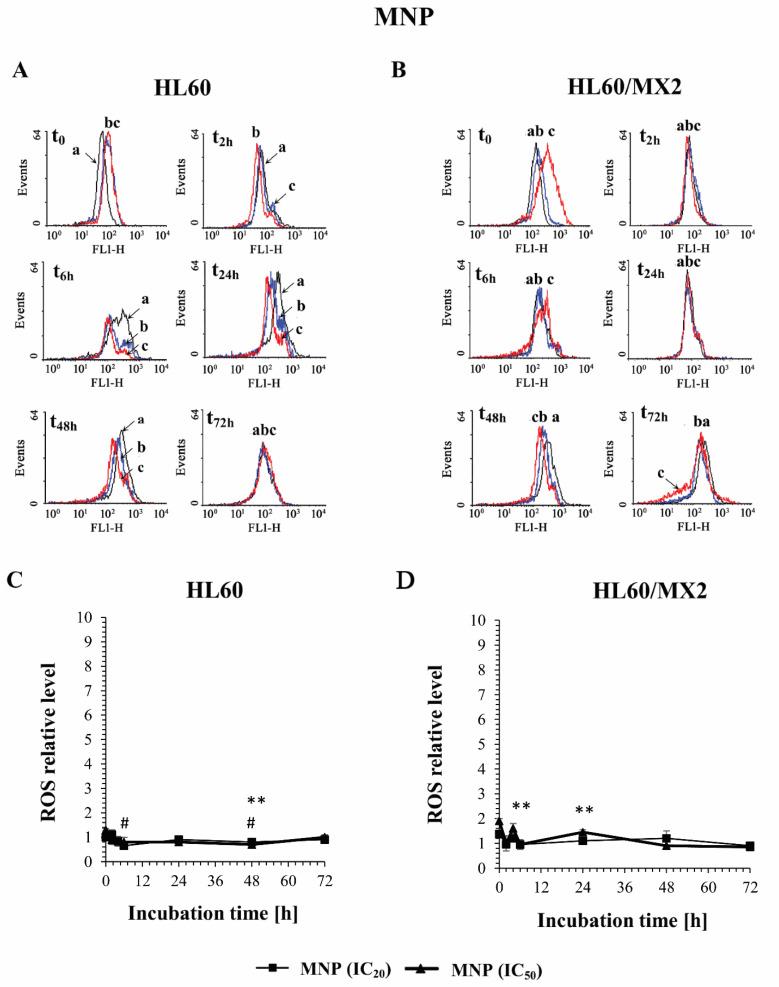
The effect of MNP on the reactive oxygen species (ROS) level in sensitive HL60 (**A**,**C**) and resistant HL60/MX2 (**B**,**D**) leukaemia cells. The cells were incubated at 37 °C with MNP used at IC_20_ (7.5 µM for HL60 cells and 7.2 µM for HL60/MX2 cells) or at IC_50_ (24.3 µM for HL60 cells and 20.5 µM for HL60/MX2 cells), respectively, up to 72 h. The ROS level was determined by flow cytometry with the use of 2′,7′-dichlorofluorescein diacetate (2′,7′-DCF-DA). The green fluorescence (FL-1 channel) of 5 × 10^3^ events per sample was measured with the use of a laser beam at 530 ± 15 nm (λ_ex_ = 488 nm). Presented histograms (**A**,**B**) are representative examples observed for control and treated cell samples (a, black—control cells; b, blue—cells treated with MNP at IC_20_; and c, red—cells treated with MNP at IC_50_). The experiment was repeated at least four times (made in triplicate) and the data are given as the mean ± SD values (**C**,**D**). The significance level of the differences observed (Student’s *t*-test) between values found for cells treated with MNP at IC_20_ (^#^ *p* < 0.05) or IC_50_ (** *p* < 0.01), respectively, compared with values found for control cells.

**Figure 3:**
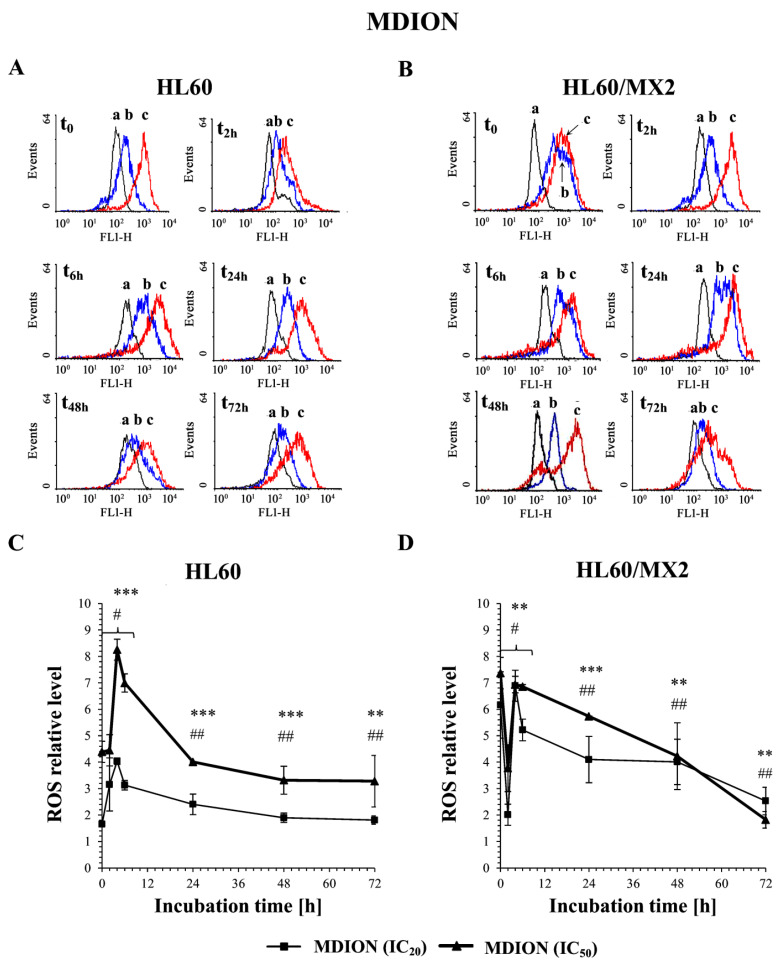
The effect of MDION on the reactive oxygen species (ROS) level in sensitive HL60 (**A**,**C**) and resistant HL60/MX2 (**B**,**D**) leukaemia cells. The cells were incubated at 37 °C with MDION used at IC_20_ (15.0 µM for HL60 cells and 17.0 µM for HL60/MX2 cells) or at IC_50_ (80.5 µM for HL60 cells and 95.5 µM for HL60/MX2 cells), respectively, up to 72 h. The ROS level was determined by flow cytometry with the use of 2′,7′-dichlorofluorescein diacetate (2′,7′-DCF-DA). The green fluorescence (FL-1 channel) of 5 × 10^3^ events per sample was measured with the use of a laser beam at 530 ± 15 nm (λ_ex_ = 488 nm). Presented histograms (**A**,**B**) are representative examples observed for control and treated cell samples (a, black—control cells; b, blue—cells treated with MDION at IC_20_; and c, red—cells treated with MDION at IC_50_). The experiment was repeated at least four times (made in triplicate) and the data are given as the mean ± SD values (**C**,**D**). The significance level of the differences observed (Student’s *t*-test) between values found for cells treated with MNP at IC_20_ (^#^ *p* < 0.05; ^##^ *p* < 0.01) or IC_50_ (** *p* < 0.01; *** *p* < 0.001), respectively, compared with values found for control cells.

Figure 2A,B and Figure 3A,B show representative histograms obtained for the control and treated HL60 and HL60/MX2 cell samples. It was found that MNP used at IC_20_ and IC_50_ caused only a slight decrease in the ROS level in sensitive HL60 cells observed from 2 h to 48 h of the experiment. During the continued long-time incubation of cells with this compound for up to 72 h, no statistically significant changes in the ROS level of examined HL60 cells were observed (Figure 2A,C). It was also found that MNP used at IC_20_ did not considerably change the ROS level in HL60/MX2 cells (Figure 2B,D). In contrast, MNP used at IC_50_ caused a noticeable rise in the ROS level in these resistant cells in the first 24 h of the experiment (to the relative level of about 1.5–2 in comparison to the untreated HL60/MX2 cells) (Figure 2B,D). Interestingly, in the case of MDION, a high stimulation of ROS generation was observed in sensitive HL60 and resistant HL60/MX2 cells (Figure 3). MDION used at IC_20_ or IC_50_ caused about a 4-fold and 8-fold increase in the ROS level, respectively, in HL60 cells in 2 h of the experiment. During continuous long-term incubation of these cells with MDION, a decrease in the ROS level was observed (approximately to the level 2-fold and 4-fold higher in the case of cells treated with this compound at IC_20_ and IC_50_, respectively, in comparison to the ROS level found for control HL60 cells) (Figure 3A,C). Similar results were also obtained for resistant HL60/MX2 cells treated with MDION used at IC_20_ and IC_50_. It caused a strong (about 6–7 fold) increase in the ROS level at both concentrations tested at the beginning of the experiment. During long-term incubation of these cells with MDION for up to 72 h, a continuous decrease in the ROS level was observed (approximately to a level 2-fold higher compared to the ROS level observed for control HL60/MX2 cells at the end of the experiment) (Figure 3B,D).

### 2.3. The Effect of MNP and MDION on Cell Cycle Distribution of Sensitive HL60 and Resistant HL60/MX2 Leukaemia Cells

**Figure 4:**
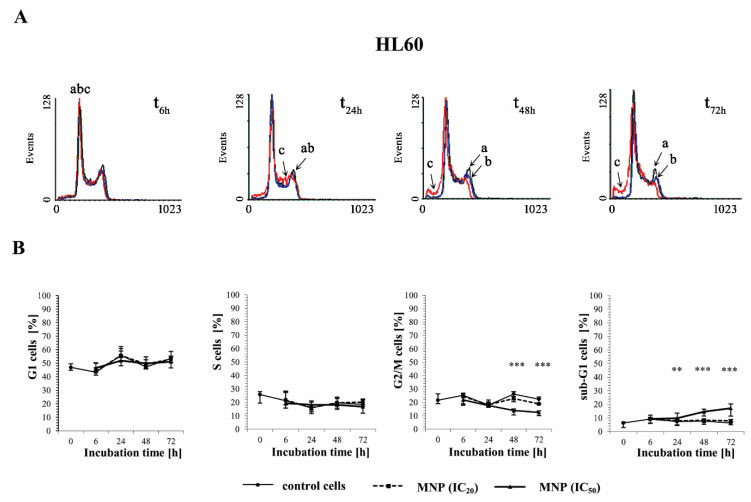

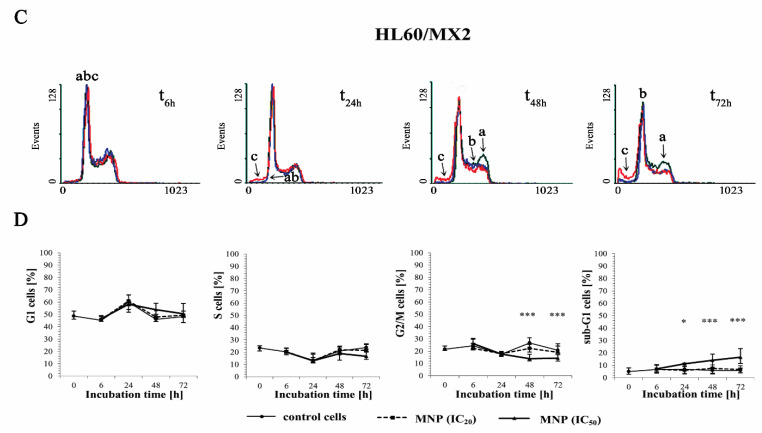
The effect of MNP on the cell cycle distribution in sensitive HL60 (**A**,**B**) and resistant HL60/MX2 (**C**,**D**) leukaemia cells and the appearance of subdiploid (sub-G1) cells with degradated DNA. The cells were incubated at 37 °C with MNP used at IC_20_ (7.5 µM for HL60 cells and 7.2 µM for HL60/MX2 cells) or at IC_50_ (24.3 µM for HL60 cells and 20.5 µM for HL60/MX2 cells), respectively, up to 72 h. The cell cycle distribution was analysed by flow cytometry (FACScan; Becton Dickinson). The red fluorescence (FL-2) of 10^4^ events per sample was measured with the use of a laser beam at 585 ± 21 nm (λ_ex_ = 488 nm) to determine the propidium iodide (PI) labelled DNA content. Presented histograms (**A**,**C**) are representative examples observed for the control and treated cell samples (a, black—control cells; b, blue—cells treated with MNP at IC_20_; and c, red—cells treated with MNP at IC_50_). The experiment was repeated at least four times (made in triplicate) and the data are given as median (interquartile range) values (**B**,**D**). The significance level of the differences observed (Mann–Whitney *U* test) between values found for cells treated with MNP at IC_50_ (* *p* < 0.05; ** *p* < 0.01; *** *p* < 0.001) compared with values found for control cells.

**Figure 5:**
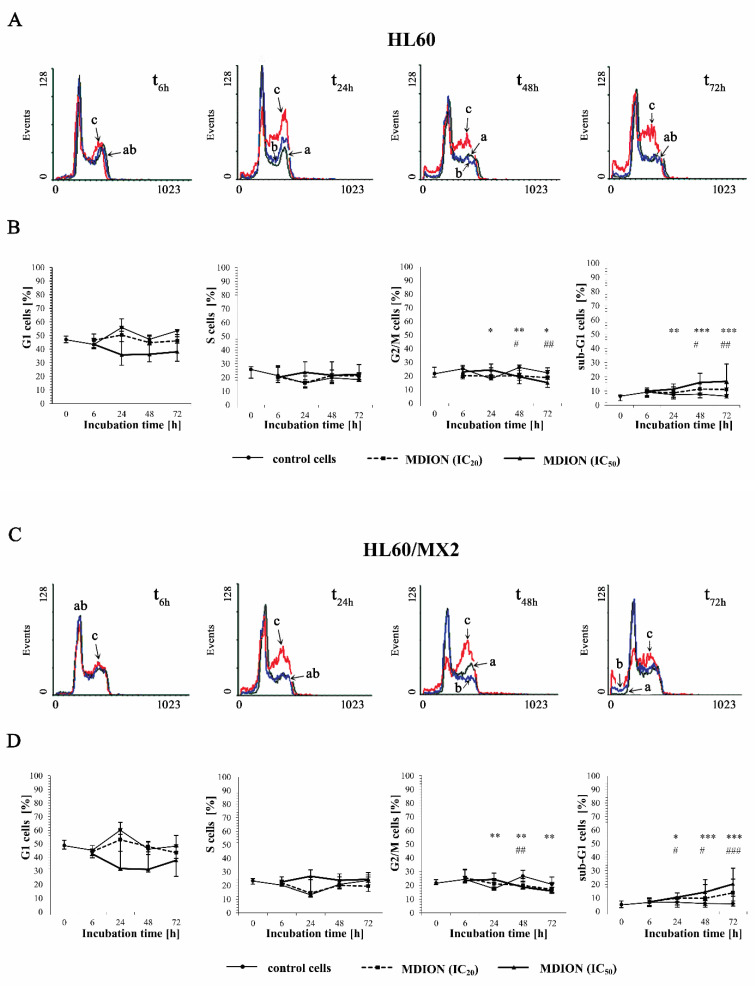
The effect of MDION on the cell cycle distribution in sensitive HL60 (**A**,**B**) and resistant HL60/MX2 (**C**,**D**) leukaemia cells and the appearance of subdiploid (sub-G1) cells with degradated DNA. The cells were incubated at 37 °C with MDION used at IC_20_ (15.0 µM for HL60 cells and 17.0 µM for HL60/MX2 cells) or at IC_50_ (80.5 µM for HL60 cells and 95.5 µM for HL60/MX2 cells), respectively, up to 72 h. The cell cycle distribution was analysed by flow cytometry (FACScan; Becton Dickinson). The red fluorescence (FL-2) of 10^4^ events per sample was measured with the use of a laser beam at 585 ± 21 nm (λ_ex_ = 488 nm) to determine the propidium iodide (PI) labelled DNA content. Presented histograms (**A**,**C**) are representative examples observed for control and treated cell samples (a, black—control cells; b, blue—cells treated with MDION at IC_20_; and c, red—cells treated with MDION at IC_50_). The experiment was repeated at least four times (made in triplicate) and the data are given as median (interquartile range) values (**B**,**D**). The significance level of the differences observed (Mann–Whitney *U* test) between values found for cells treated with MDION at IC_20_ (^#^ *p* < 0.05; ^##^ *p* < 0.01; ^###^ *p* < 0.001) or IC_50_ (* *p* < 0.05; ** *p* < 0.01; *** *p* < 0.001), respectively, compared with values found for control cells.

The effect of the selected pyridinium salts (MNP and MDION) on the cell cycle distribution of sensitive HL60 and resistant HL60/MX2 leukaemia cells was studied by performing PI staining. The examined compounds were used at IC_20_ and IC_50_ and their effects on the cell cycle distribution of HL60 and HL60/MX2 cells were observed up to 72 h of incubation. Figure 4A,C and Figure 5A,C show representative histograms obtained for control and treated HL60 and HL60/MX2 cell samples.

It was found that the cell cycle distribution and the percentage of the sub-G1 subpopulation of sensitive HL60 (Figure 4A,B) and HL60/MX2 (Figure 4C,D) cells treated with MNP used at IC_20_ did not change significantly compared to the control cell population. No statistically significant differences compared to control cells were also observed for HL60 and HL60/MX2 cells in their distribution in G1 and S phases upon treatment with MNP used at IC_50_. However, it caused a marked decrease in the percentage of G2/M subpopulations starting from 48 h of the experiment. Additionally, the incubation of sensitive HL60 and HL60/MX2 cells with this compound at IC50 had a pronounced effect on the appearance of the sub-G1 subpopulation observed from 24 h to the end of the experiment (up to 18% and 16% of the total cell population found for HL60 and HL60/MX2, respectively, in the 72 h of the experiment, whereas the percentage of sub-G1 subpopulation for both sensitive and resistant control cells was less than 5%).

In the case of MDION used at IC_20_, no significant changes were observed in the distribution of sensitive HL60 (Figure 5A,B) and resistant HL60/MX2 (Figure 5C,D) cells in G1 and S phases compared to control cells. However, it caused a slight decrease in the percentage of G2/M subpopulations observed for both sensitive and MDR cells from 48 h of the experiment. Simultaneously, the treatment of HL60 and HL60/MX2 cells with MDION used at IC_20_ resulted in a noticeable increase in the percentage of sub-G1 subpopulations (up to 10% and 15% of the total cell population for HL60 and HL60/MX2 cells, respectively, in 72 h of the experiment, whereas the percentage of sub-G1 subpopulation for both sensitive and resistant control cells was less than 5%). In the case of MDION used at a higher concentration (IC_50_), no statistically significant differences in the cell distribution in G1 and S phases compared to control cells were observed for sensitive HL60 or HL60/MX2 cells up to 72 h. However, it caused a slight decrease in the percentage of G2/M cells after 24 h of the experiment. Additionally, a significant increase in the percentage of sub-G1 subpopulations of HL60 and HL60/MX2 cells (up to 16% and 20%, respectively) was also observed.

### 2.4. The Effect of MNP and MDION on Cellular DNA Damage in Sensitive HL60 and Resistant HL60/MX2 Leukaemia Cells

Cellular DNA damage was assessed by flow cytometry with the use of the γ-H2AX histone phosphorylation assay and γ-anti-H2AX antibody conjugated with AlexaFluor 488 since H2AX phosphorylation is a well-recognised biomarker of double strand breaks (DSBs). The effect of MNP and MDION on cellular DNA damage in sensitive HL60 and resistant HL60/MX2 cells was examined up to 24 h of incubation. Additionally, the cells treated with anthracycline antitumour drug, IDA, which exhibits a well-proven ability to induce DSB damage in cancer cells, were taken as a reference positive control [26].

**Figure 6:**
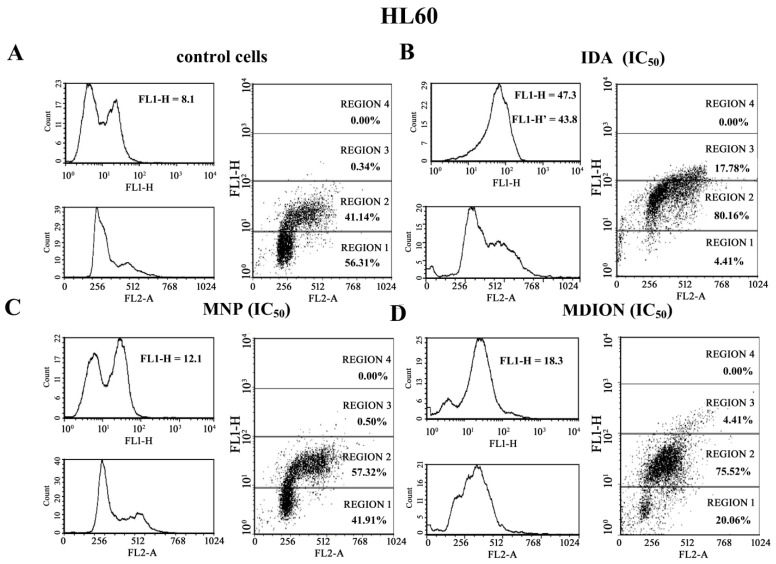

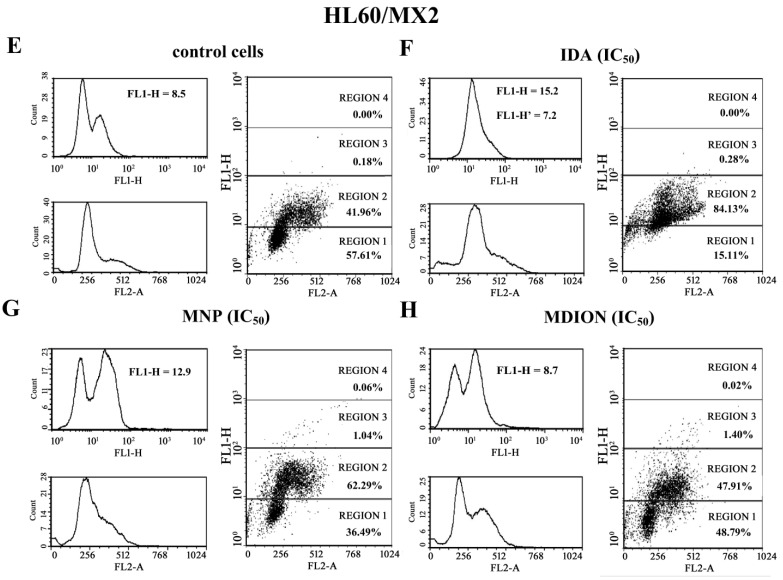
Level of the phosphorylated form of γ-H2AX in sensitive HL60 cells (**A**–**D**) and resistant HL60/MX2 (**E**–**H**) leukaemia cells treated with idarubicin (IDA; positive control), MNP, and MDION. The cells were incubated at 37 °C with studied compounds used at IC_50_ (in the case of IDA, at 9.5 µM for HL60 cells and at 19.0 µM for HL60/MX2 cells, in the case of MNP, at 24.3 µM for HL60 cells and at 20.5 µM for HL60/MX2 cells, in the case of MDION, at 80.5 µM for HL60 cells and at 95.5 µM for HL60/MX2 cells) for 24 h. The experimental procedure for measuring the cellular level of the phosphorylated form of γ-H2AX is described in detail in the Materials and methods section. The green fluorescence FL1-H and red fluorescence FL2-A of 5 × 10^3^ single events per sample were measured with the use of a laser beam at 530 ± 30 nm (λ_ex_ = 488 nm) or 585 ± 21 nm (λ_ex_ = 488 nm), respectively. The figure shows representative histograms and dot-plots obtained for control and treated cell samples in the presence of propidium iodide (PI) to determine the DNA content (FL2-A) and AlexaFluor 488-conjugated monoclonal antibody staining the phosphorylated form of histone γ-H2AX (FL1-H; FL1-H’ represent data obtained after subtracting the value corresponding to the IDA autofluorescence signal).

**Figure 7:**
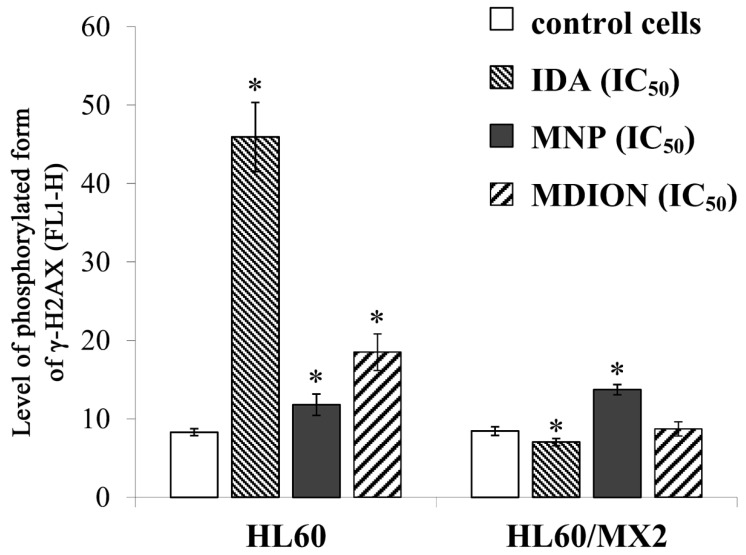
The DSB formation in sensitive HL60 and resistant HL60/MX2 leukaemia cells treated with idarubicin (IDA; positive control), MNP, and MDION. The cells were incubated at 37 °C with studied compounds used at IC_50_ (in the case of IDA, at 9.5 µM for HL60 cells and at 19.0 µM for HL60/MX2 cells, in the case of MNP, at 24.3 µM for HL60 cells and at 20.5 µM for HL60/MX2 cells, in the case of MDION, at 80.5 µM for HL60 cells and at 95.5 µM for HL60/MX2 cells) for 24 h. AlexaFluor 488-conjugated monoclonal antibody staining of the phosphorylated form of histone γ-H2AX reflecting the level of DSB damage of cellular DNA was done for control and cell samples treated with studied compounds at IC_50_. The experimental procedure for performing anti-γ-H2AX labelling is described in detail in the Materials and methods section. The green fluorescence FL1-H of 5 × 10^3^ single events per sample was measured with the use of a laser beam at 530 ± 30 nm (λex = 488 nm). The level of the phosphorylated form of γ-H2AX was determined by recording the FL1-H values reflecting the signal of AlexaFluor 488 conjugated to the γ-anti-H2AX antibody. The data represent the mean ± SD values of three independent experiments (made in duplicate). The significance level of the differences observed (Student’s t-test) between values found for cells treated with IDA, MNP, or MDION at IC_50_, respectively, (* *p* < 0.05), compared with values found for control cells.

Figure 6 shows representative histograms and cytograms obtained for control and treated sensitive HL60 and resistant HL60/MX2 cell samples (FL1-H reflects the signal of AlexaFluor 488 conjugated to the γ-anti-H2AX antibody, whereas FL2-A represents the signal of PI being the marker of the cellular DNA content). It was found that the reference compound, IDA used at IC_50_ (9.5 µM for HL60 cells and 19.0 µM for HL60/MX2 cells), provoked a high rise (about 5.4-fold) in the level of DSBs in sensitive HL60 cells (Figure 6A,B and Figure 7), whereas the examined pyridinium salts, MNP and MDION, respectively, used at IC_50,_ caused much smaller increase (about 1.5-fold and 2.3-fold, respectively) in cellular DNA damage compared to untreated control HL60 cells (Figure 6C,D and Figure 7). Interestingly, in the case of resistant HL60/MX2 cells, it was found that IDA (IC_50_) and MDION (IC_50_) were not able to induce DSB damage (Figure 6F,H and Figure 7). A slight increase (about 1.5-fold) in the level of γ-H2AX-positive staining was only observed for HL60/MX2 cells treated with MNP (IC_50_) (Figure 6G and Figure 7).

### 2.5. The Effect of MNP and MDION on Caspase-3 and Caspase-8 Activation in Sensitive HL60 and Resistant HL60/MX2 Leukaemia Cells

To evaluate the ability of MNP and MDION to trigger programmed cell death occurring with the participation of caspase-3 and caspase-8, sensitive HL60 and resistant HL60/MX2 cells were incubated for 72 h with these compounds used at IC_50_ and IC_90_ and subsequently, the activities of these caspases were determined with the aid of the FLICA test.

**Figure 8:**
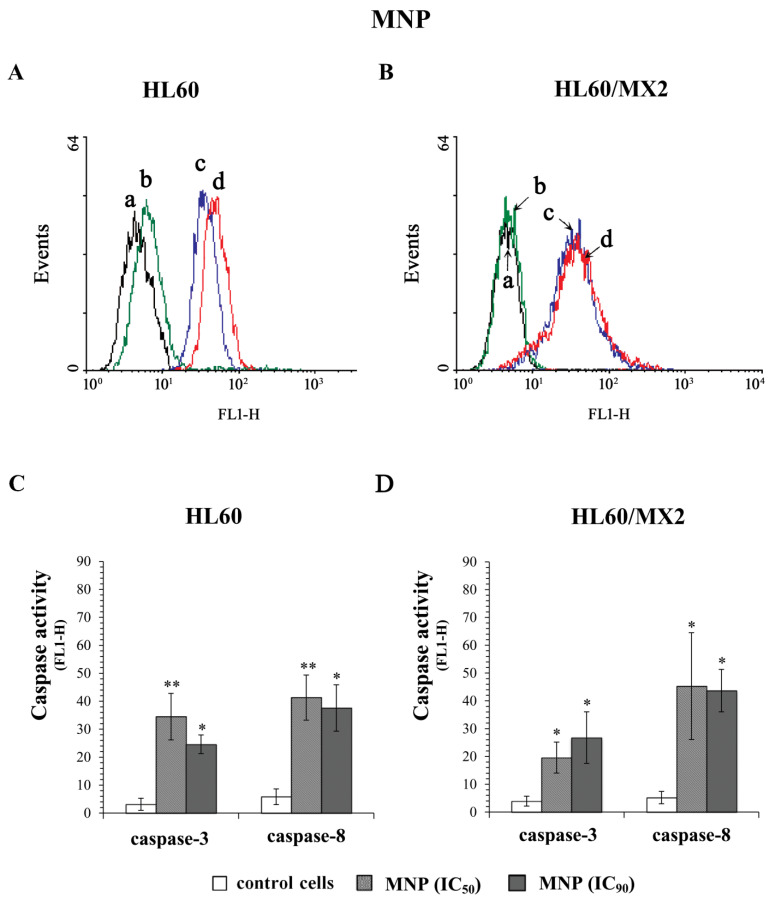
Caspase-3 and caspase-8 activity in sensitive HL60 (**A**,**C**) and resistant HL60/MX2 (**B**,**D**) leukaemia cells treated with MNP. The cells were incubated at 37 °C with MNP at IC_50_ (24.3 µM for HL60 cells and 20.5 µM for HL60/MX2 cells) or at IC_90_ (82.1 µM for HL60 cells and 77.3 µM for HL60/MX2 cells), respectively, for 72 h. The activity of caspases was determined using specific membrane-permanent, fluorescent inhibitor-based FLICA caspase probes (FITC-DEVD-FMK for caspase-3 and FITC-IETD-FMK for caspase-8, respectively). Presented histograms (**A**,**B**) are representative examples observed for control and treated cell samples (a, black—caspase-3 in control cells; b, green—caspase-8 in control cells; c, blue—caspase-3 in cells treated with MNP at IC_90_; and d, red—caspase-8 in cells treated with MNP at IC_90_). The experiment was repeated at least three times (made in duplicate) and the data are given as the mean ± SD values (**C**,**D**). The significance level of the differences observed (Student’s *t*-test) between values found for cells treated with MNP at IC50 or IC90, respectively, (* *p* < 0.05; ** *p* < 0.01) compared with values found for control cells.

**Figure 9:**
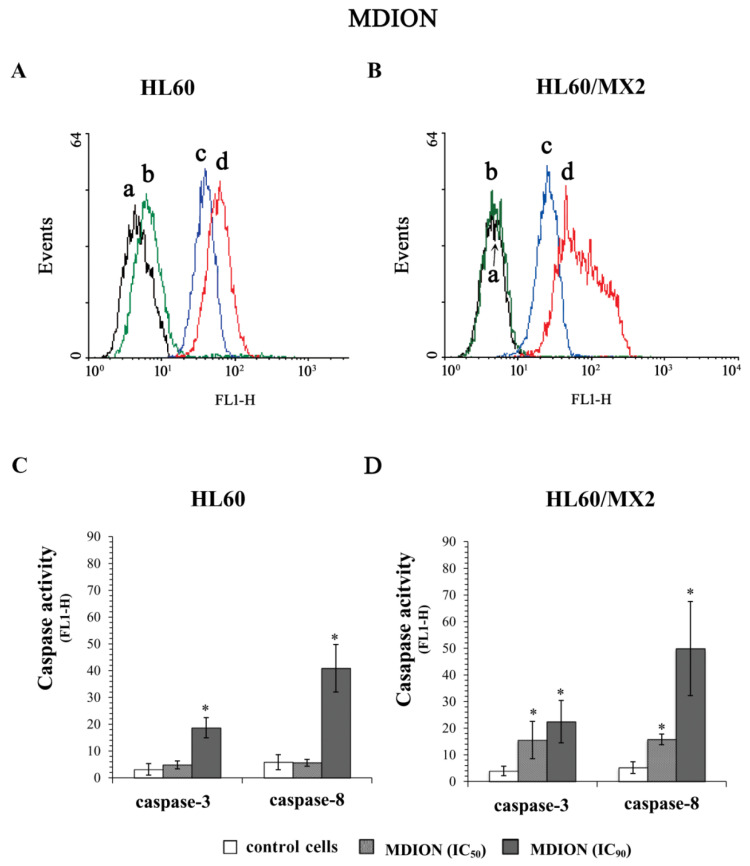
Caspase-3 and caspase-8 activity in sensitive HL60 (**A**,**C**) and resistant HL60/MX2 (**B**,**D**) leukaemia cells treated with MDION. The cells were incubated at 37 °C with MDION used at IC_50_ (80.5 µM for HL60 cells and 95.5 µM for HL60/MX2 cells) or at IC_90_ (573.0 µM for HL60 cells and 591.2 µM for HL60/MX2 cells), respectively, for 72 h. The activity of caspases was determined using specific membrane-permanent, fluorescent inhibitor-based FLICA caspase probes (FITC-DEVD-FMK for caspase-3 and FITC-IETD-FMK for caspase-8, respectively). Presented histograms (**A**,**B**) are representative examples observed for control and treated cell samples (a, black—caspase-3 in control cells; b, green—caspase-8 in control cells; c, blue—caspase-3 in cells treated with MDION at IC_90_; and d, red—caspase-8 in cells treated with MDION at IC_90_). The experiment was repeated at least three times (made in duplicate) and the data are given as the mean ± SD values (**C**,**D**). The significance level of the differences observed (Student’s *t*-test) between values found for cells treated with MDION at IC_50_ or IC_90_, respectively, (* *p* < 0.05) compared with values found for control cells.

Figure 8A,B and Figure 9A,B show representative histograms obtained for control and treated HL60 and HL60/MX2 cell samples. As it was presented in Figure 8, MNP used at IC_50_ and IC_90_ caused a very high (5–12-fold) increase in the activity of both caspase-3 and caspase-8 in sensitive HL60 (Figure 8A,C) and resistant HL60/MX2 (Figure 8B,D) cells. However, in the case of sensitive HL60 cells treated with MDION used at IC_50_, the relative activities of these caspases were comparable with the activity levels observed in control cells (Figure 9A,C). Nevertheless, at the higher concentration (IC_90_) used, this compound was able to induce the activation of both caspase-3 (about 7-fold) and caspase-8 (about 8-fold) in sensitive HL60 cells. In the case of resistant HL60/MX2 cells, MDION used at IC_50_, and IC_90_ provoked an important (3–10-fold) augmentation in both caspase-3 and caspase-8 activities (Figure 9B,D).

### 2.6. The Effect of MNP and MDION on Lysosomal Membrane Integrity in Sensitive HL60 and Resistant Leukaemia HL60/MX2 Cells

To evaluate the involvement of MNP and MDION on lysosomal membrane permeabilization (LMP) and its potential role in inducing the programmed death of sensitive HL60 and resistant HL60/MX2 cells, lysosomal integrity assays were performed with the use of the fluorescent acidotropic probe LysoTracker Green DND-26 which accumulates in intact lysosomes. The cells were incubated with MNP and MDION used at IC_20_ and IC_50_, respectively, for up to 72 h and the probe accumulation measurements were performed in a function of time (at 30 min, 2, 6, 24, 48, and 72 h of the experiment duration).

**Figure 10:**
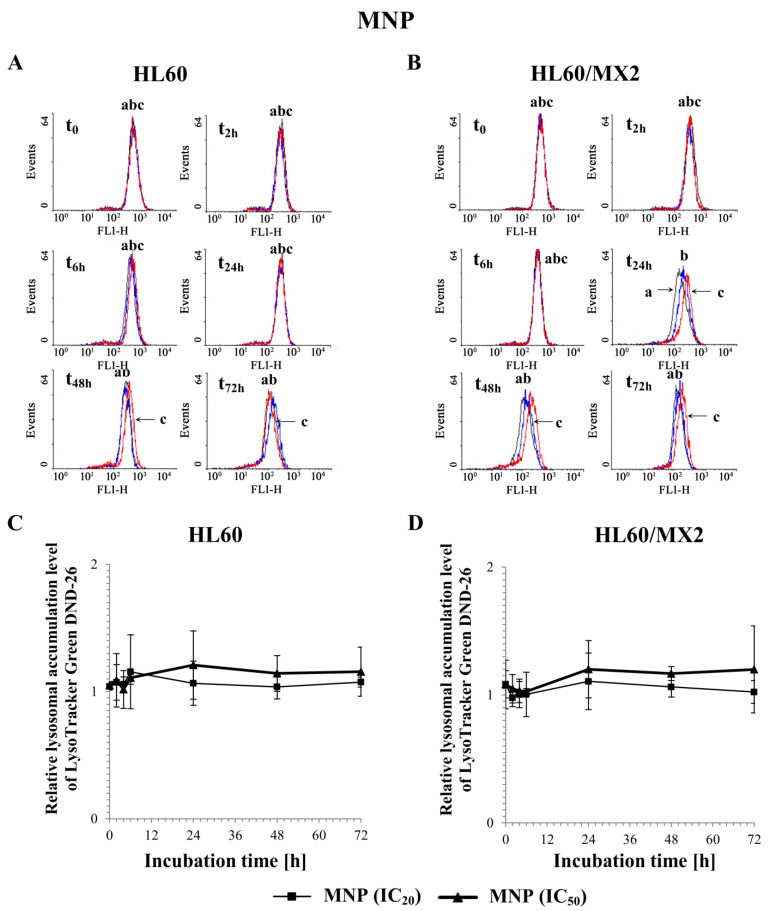
The effect of MNP on lysosomal integrity in sensitive HL60 (**A**,**C**) and resistant HL60/MX2 (**B**,**D**) leukaemia cells. The cells were incubated at 37 °C with MNP used at IC_20_ (7.5 µM for HL60 cells and 7.2 µM for HL60/MX2 cells) or at IC_50_ (24.3 µM for HL60 cells and 20.5 µM for HL60/MX2 cells), respectively, up to 72 h. The lysosomal integrity of the cells was examined with the use of flow cytometry by measuring the level of LysoTracker Green DND-26, a specific fluorescent lysosomal probe. The green fluorescence (FL-1 channel) of 5 × 10^3^ events per sample was measured with the use of a laser beam at 530 ± 15 nm (λ_ex_ = 488 nm). Presented histograms (**A**,**B**) are representative examples observed for control and treated cell samples (a, black—control cells; b, blue—cells treated with MNP at IC_20_; and c, red—cells treated with MNP at IC_50_). The experiment was repeated at least four times (made in triplicate) and the data are given as the mean ± SD values (**C**,**D**). Statistical analysis of the differences between values found for cells treated with MNP at IC_20_ or IC_50_ was carried out using Student’s *t*-test (no statistically significant changes were found, *p* > 0.05).

**Figure 11:**
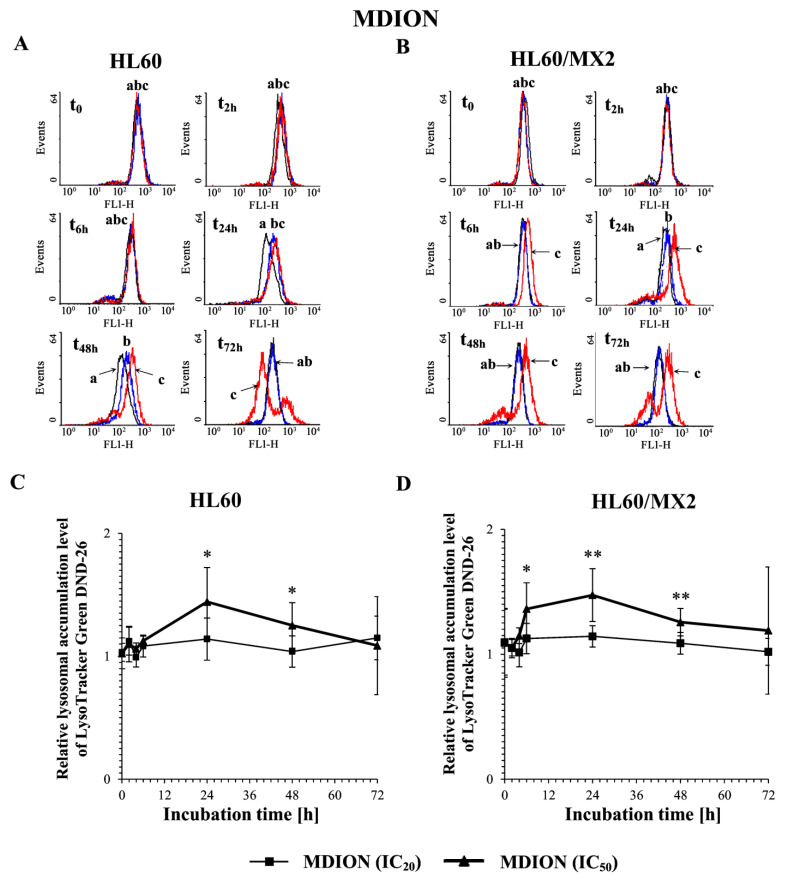
The effect of MDION on lysosomal integrity in sensitive HL60 (**A**,**C**) and resistant HL60/MX2 (**B**,**D**) leukaemia cells. The cells were incubated at 37 °C with MDION used at IC_20_ (15.0 µM for HL60 cells and 17.0 µM for HL60/MX2 cells) or at IC_50_ (80.5 µM for HL60 cells and 95.5 µM for HL60/MX2 cells), respectively, up to 72 h. The lysosomal integrity of the cells was examined with the use of flow cytometry by measuring the level of LysoTracker Green DND-26, a specific fluorescent lysosomal probe. The green fluorescence (FL-1 channel) of 5 × 10^3^ events per sample was measured with the use of a laser beam at 530 ± 15 nm (λ_ex_ = 488 nm). Presented histograms (**A**,**B**) are representative examples observed for control and treated cell samples (a, black—control cells; b, blue—cells treated with MDION at IC_20_; and c, red—cells treated with MDION at IC_50_). The experiment was repeated at least four times (made in triplicate) and the data are given as the mean ± SD values (**C**,**D**). The significance level of the differences (Student’s *t*-test) between values found for cells treated with MDION at IC_20_ (* *p* < 0.05) or IC_50_ (* *p* < 0.05; ** *p* < 0.01), respectively, compared with values found for control cells.

Figure 10A,B and Figure 11A,B show representative histograms obtained for control and treated sensitive HL60 and resistant HL60/MX2 cell samples. It was found that MNP used at IC_20_ and IC_50_ did not cause the lysosomal membrane permeabilization in studied sensitive HL60 (Figure 10A,C) and resistant HL60/MX2 (Figure 10B,D) cells (the LysoTracker Green DND-26 accumulation found for cells treated with this pyridinium salt was almost the same compared with the accumulation level observed for control cells). Similar results were obtained for sensitive HL60 cells (Figure 11A,C) and their MDR counterparts (Figure 11B,D) treated with MDION used at IC_20_, whereas this compound used at higher concentration (IC_50_) caused a marked increase in LysoTracker Green DND-26 probe accumulation (to the relative level of about 1.5 compared with the accumulation level observed for control cells in 24 h of the conducted experiment), indicating an important alteration in the LMP of these cells upon MDION treatment. However, during the continued long-time incubation of sensitive HL60 and resistant HL60/MX2 cells with this compound used at IC_50_, the decrease in the accumulation level of this acidotropic dye was observed up to levels characteristic for control cells at the end of the experiment.

## 3. Discussion

Multidrug resistance (MDR) of tumour cells to conventional chemotherapeutics constitutes one of the major clinical problems causing the failure of anticancer therapy [4]. Therefore, the search for new drugs that remain efficient when treating MDR tumours is of high importance [1,2,3]. This is challenging research that must take into account the multifactorial character of the MDR phenomenon [4]. Among the promising agents in this search are compounds having scaffolds containing a pyridine moiety or other pyridine derivatives, including pyridinium salts, MNP and MDION [15,19,21,23]. In our previous study, it was demonstrated that these agents retain high efficacy against MDR leukaemia HL60/VINC and HL60/DOX cells overexpressing ABC drug efflux transporters (P-gp and MRP1, respectively) [23]. However, up to now, very little is known about their activity and cellular mechanisms of action in tumour cells exhibiting other phenotypes of MDR. Therefore, in this study, we examined in a comparative manner the effect of MNP and MDION on sensitive HL60 cells and their MDR counterparts, namely HL60/MX2, exhibiting the MDR phenotype related to the presence of the mutated α isoform of topoisomerase II and the lack of the β isoform of this enzyme.

It was found that MNP and MDION exhibited very similar activities to inhibit the growth of the studied sensitive HL60 and resistant HL60/MX2 cells. The resistance factor (RF) values determined for these compounds were close to 1.0. However, it was found that MNP was several times more active than MDION towards sensitive HL60 cells and their MDR counterparts. Similar differences in their antileukaemic efficacy were also reported earlier in the case of resistant HL60/VINC and HL60/DOX cells [23].

It is proposed that antitumour activity of some bioreductive agents is associated with their ability to trigger oxidative stress, related to ROS generation [27,28]. It was also reported previously that MNP and MDION are able to shift the NAD(P)H/NAD(P)^+^ equilibrium in non-enzymatic and enzyme-dependent processes involving two-electron and one-electron transfer reactions [22,23,24,25]. Therefore, in this work, the effect of these pyridinium salts on the cellular ROS level was evaluated. It was found that MNP and MDION modulated the level of ROS in sensitive HL60 and resistant HL60/MX2 cells in a dose-dependent and time-dependent manner. MNP used at IC_20_ and IC_50_ caused only a slight increase in the ROS level of sensitive HL60 cells. In contrast, this agent used at IC_50_ caused a noticeable augmentation in the ROS level in resistant HL60/MX2 cells in the first 24 of the experiment (to the relative level of about 1.5–2 in comparison to the control HL60/MX2 cells). Interestingly, MDION used both at IC_20_ or IC_50_ was highly active in stimulating ROS generation in sensitive HL60 and resistant HL60/MX2 cells in the first hours of the performed experiments (about a 4–8-fold increase in the ROS level was observed for HL60 and HL60/MX2 cells).

An increasing body of evidence from experimental data indicates that the deregulation of cellular oxidoreductive balance by xenobiotics may be a crucial factor that triggers mechanisms causing the cell division arrest of cancer cells and initiation of signalling pathways leading to the programmed cell death [29,30]. Therefore, the effect of MNP and MDION on cell cycle distribution was subsequently investigated. It was found that these compounds used at IC_20_ and IC_50_ caused dose-dependent and time-dependent alterations in the cell cycle of sensitive HL60 and resistant HL60/MX2 cells, resulting in the decrease in the percentage of the cells in G2/M phases and the appearance of the sub-G1 subpopulation. However, they did not provoke any cell cycle arrest at the G1/S or G2/M control points in studied sensitive HL60 cells and their MDR counterparts (HL60/MX2 cells). The abrogation of the effective G1/S checkpoint in these cells under treatment with studied compounds could be related to the lack of a functional p53 protein [31,32]. Furthermore, the results obtained with the use of the γ-H2AX histone phosphorylation assay showed that, in contrast to the reference compound—IDA (IC_50_), the examined pyridinium salts, MNP (IC_50_) and MDION (IC_50_), caused only a small increase (about 1.5-fold and 2.3-fold, respectively) in the level of DSBs in sensitive HL60 cells. Interestingly, in the case of their resistant topoisomerase II-defective HL60/MX2 counterparts, it was found that IDA (IC_50_) and MDION (IC_50_) were not able to induce cellular DNA damage. A slight increase (about 1.5-fold) in the level of DSBs was observed only for HL60/MX2 cells treated with MNP (IC_50_). Thus, the results obtained in the study indicate that the induction of DSBs by MNP and MDION was not a significant mechanism of action of these pyridinium salts in sensitive HL60 and resistant HL60/MX2 cells.

In the next part of the study, the effect of MNP and MDION on cell death of sensitive HL60 cells and their MDR counterparts (HL60/MX2 cells) was investigated. The obtained results showed that the examined pyridinium salts used at IC_50_ and IC_90_ were highly active in triggering caspase-dependent apoptotic death not only of sensitive HL60 cells but also their MDR counterparts (HL60/MX2 cells). It was found that MNP and MDION provoked a significant activation of caspase-3 and caspase-8 in studied sensitive and resistant HL60 cells. However, for deciphering whether these agents triggered the apoptotic death of examined cells with the involvement of only the extrinsic death-receptor mediated death pathway or also the intrinsic mitochondrial signalling, further studies are required to evaluate the expression and/or activation of mediators involved in specific apoptosis pathways, such as the death receptors, cellular factors belonging to the Bcl family, and the release of mitochondrial cytochrome c to the cytoplasm and the activation of procaspase-9.

In recent years, there has been a growing number of reports showing that some antitumour compounds, including agents that stimulate cellular ROS generation leading to oxidative stress, are able to trigger lysosomal cell death pathways [33,34]. Their activity in promoting non-apoptotic lysosomal cell death of tumour cells is often due to the increase in lysosomal membrane permeabilization (LMP), leading to the release to the cytoplasm of lysosomal contents, mainly cathepsins involved in cell death signal cascades [35,36]. The concomitant induction of apoptotic caspase-dependent and lysosomal-dependent death pathways of sensitive HL60 cells and their MDR counterparts (HL60/VINC and HL60/DOX) has been previously reported for anthrapyridone CO1 [37]. In contrast, the results of our recent study performed with the use of sensitive HL60 cells and resistant HL60/VINC and HL60/MX2 cells showed that plant phenolic acids, gallic acid (GA) and ellagic acid (EA), induced the programmed death of these cells occurring without the participation of lysosomes [38]. The ability of MNP and MDION to target lysosomes of studied HL60 and HL60/MX2 cells was examined with the aid of LysoTracker Green DND-26, which is a specific fluorescent lysosomal probe. It was found that MNP used at IC_20_ and IC_50_ did not cause the permeabilization of the lysosomal membrane of examined cells. In contrast, MDION used at IC_50_ affected lysosomes of sensitive HL60 and resistant HL60/MX2 cells in a time-dependent manner, suggesting that these organelles could also be involved in triggering the programmed death of studied sensitive and MDR leukaemia HL60 cells by MDION, in addition to inducing caspase-dependent apoptosis. Surprisingly, unlike many other lysosome-targeting anticancer drugs increasing LMP [35,36,37], it caused a temporary marked raise in LysoTracker Green DND-26 probe accumulation, suggesting that this pyridinium salt affects lysosomes of studied leukaemia HL60 and HL60/MX2 cells in another way. Therefore, further investigations are needed to elucidate the impact of MDION on lysosome functions and the role of these organelles in the death of sensitive HL60 cells and their MDR counterparts induced by MDION. Moreover, the interplay between apoptotic and lysosomal players participating in the activation and/or amplification of programmed cell death signalling [39,40] of studied cells triggered by this compound remains to be identified.

Up to now, the findings of the present study allow us to propose a putative model of MNP and MDION action in sensitive leukaemia HL60 and resistant HL60/MX2 cells possessing the mutated α isoform of topoisomerase II and lacking the β isoform of this enzyme, as presented below.

The examined pyridinium salts cause very quick changes in the intracellular ROS level, occurring in a dose-dependent manner, followed by provoking some important cell cycle disturbances and the appearance of a sub-G1 subpopulation; ultimately leading to the induction of programmed cell death. However, it is worth emphasising that the induction of DSB formation by MNP and MDION is not a significant mechanism of action of these pyridinium salts in sensitive HL60 and resistant HL60/MX2 cells. Both studied compounds trigger apoptosis of sensitive HL60 cells and their MDR counterparts with the involvement of caspase-3 and caspase-8. Additionally, the results found for MDION, highly active in stimulating ROS production, indicate that lysosomes may also be involved in the programmed death of sensitive HL60 and resistant HL60/MX2 leukaemia cells induced by this agent.

## 4. Materials and Methods

### 4.1. Chemicals

1-Methyl-3-nitropyridine chloride (MNP) and 3,3,6,6,10-pentamethyl-3,4,6,7-tetrahydro-[1,8(*2H,5H*)-dion]acridine chloride (MDION) were synthesised according to the procedure published elsewhere [22,25]. Idarubicin (IDA), 3-(4,5-dimethylthiazolyl-2)-2,5-diphenyltetrazolium bromide (MTT), 2′,7′-dichlorofluorescein diacetate (2′,7′-DCF-DA), ribonuclease A (RNase A), propidium iodide (PI), dimethyl sulfoxide (DMSO), and dodecyl sulfate sodium (SDS) were purchased from Sigma-Aldrich (Saint Louis, MO, USA). The CaspGLOW™ Fluorescein Active Caspase-3 Staining Kit and CaspGLOW™ Fluorescein Active Caspase-8 Staining Kit were purchased from eBioscience, LysoTracker Green DND-26 from Molecular Probes (Eugene, OR, USA), N,N-dimethylformamide (DMF) from Merck, and anti-phospho-H2AX antibody conjugated with Alexa 488 from Becton Dickinson Biosciences (Franklin Lakes, NY, USA).

### 4.2. Cell Culture

The HL60 human promyelocytic leukaemia cell line was obtained from the Division of Biology, Kansas State University, Manhattan, Kansas, USA, and its resistant HL60/MX2 subline was obtained from the Department of Medicine, University of Utah School of Medicine, Salt Lake City, UT, USA. The cells were grown in RPMI 1640 medium supplemented with 2 mM glutamine, 10% foetal bovine serum (FBS), 100 U/mL penicillin, and 100 µg/mL streptomycin (Gibco Limited, Grand Island, NY, USA) at 37 °C in a humidified atmosphere of 95% air and 5% CO_2_. All cultures (HL60 and HL60/MX2) were initiated at a density of 10^5^ cells/mL and grown for 72 h to reach the steady-state growth phase. Cell viability was assessed by the trypan blue (Sigma-Aldrich, Saint Louis, MO, USA) exclusion test with the use of a Burker hemocytometer.

### 4.3. Cell Growth Inhibition Assay

The effects of MNP and MDION (dissolved in water) on cell growth were determined by incubating cells (10^4^/well) with 10 different concentrations of these compounds (varying in the range of 1–100 µM for MNP and 5–1000 µM for MDION, respectively) for 72 h at 37 °C in standard 96-well plates. The cell growth inhibition was examined by MTT reduction assay. The purple formazan crystals obtained were dissolved in a 20% SDS:DMF (1:1, v:v) mixture. The absorbance (OD570 nm) was measured with the use of a microplate reader (Biochrom Asys UVM 340, Eugendorf, Austria). To determine the percentage of cell growth inhibition, untreated cell samples were incubated in a culture medium for 72 h at 37 °C and used as a control. The IC_20_, IC_50_, and IC_90_ values, that is, MNP or MDION concentrations required to inhibit 20%, 50%, and 90% of the cell growth, respectively, were determined with the use of Microsoft Excel software.

### 4.4. Intracellular ROS Level Assay

The cells (10^5^/mL) were incubated at 37 °C in the presence of MNP or MDION used at IC_20_ or IC_50_, respectively, and at the indicated time points (0, 2, 4, 6, 24, 48, and 72 h) they were stained for 30 min with 1µM of 2′,7′-DCF-DA. Thereafter, the samples were analysed by flow cytometry (FACScan; Becton Dickinson, Franklin Lakes, NJ, USA). The green fluorescence (FL-1) of 5 × 10^3^ events per sample was measured with the use of a laser beam at 530 ± 15 nm (λ_ex_ = 488 nm). The data were analysed using BD CellQuest Pro (Becton Dickinson) as well as FSC Express, version 4.0 (DeNovo) software. In addition, control assays were carried out at 72 h to confirm 20% and 50% inhibition of cell growth by the studied compounds, respectively, by counting the viable cells in the presence of trypan blue with the use of a Burker hemocytometer.

### 4.5. Cell Cycle Distribution Analysis

The cells (10^5^/mL) were incubated at 37 °C with MNP or MDION used at IC_50_ or IC_90_, respectively, up to 72 h. At indicated incubation time points (0, 6, 24, 48, and 72 h) they were collected (5 × 10^5^ cells per sample) by centrifugation (300× *g*, 5 min, 4 °C) and washed twice with 1 mL of ice-cold phosphate-buffered saline (PBS). The resulting cell pellet was suspended in 0.5 mL PBS and fixed in 3 mL ice-cold 70% ethanol at −20 °C. Thereafter, the obtained samples containing fixed cells were centrifuged (300× *g*, 5 min, 4 °C), the pellet was rehydrated in PBS, and then centrifuged again. Thereafter, the cells were incubated with 0.2 mL PBS containing PI (20 µg/mL) and RNase A (100 µg/mL) in the dark for 30 min at room temperature. Samples were analysed by flow cytometry (FACScan; Becton Dickinson). The red fluorescence (FL-2) of 10^4^ events per sample was measured with the use of a laser beam at 585 ± 21 nm (λ_ex_ = 488 nm) to determine the DNA content. The percentage of cells in a specific phase of the cell cycle and the percentage of cells with sub-diploid DNA (sub-G1 fraction) were calculated with the use of the BD CellQuest Pro (Becton Dickinson) as well as FSC Express, version 4.0 (DeNovo) software. In addition, control assays were carried out at 72 h to confirm 50% and 90% inhibition of cell growth by the studied compounds, respectively, by counting the viable cells in the presence of trypan blue with the use of a Burker hemocytometer.

### 4.6. γ-H2AX Histone Phosphorylation Assay

The cells (10^5^/mL) were incubated at 37 °C with MNP or MDION at IC_50_ for 24 h. Additionally, the cells incubated in the same culture conditions with IDA used at IC_50_ were used as a positive control. At the indicated time point (24 h), the cells were collected by centrifugation (300× *g*, 5 min, 4 °C). Thereafter, they were washed twice with PBS. The obtained cell pellet was fixed in formaldehyde and subsequently in ice-cold 70% ethanol at −20 °C. The samples containing fixed cells were centrifuged (300× *g*, 5 min, 21 °C) and the resulting pellet was washed in PBS. Thereafter, the cells were treated for 30 min with 0.2% Triton X-100 prepared in 1% bovine serum albumin PBS solution. Then, the samples were incubated with an anti-phospho-H2AX antibody conjugated with Alexa 488 and afterward in PBS containing RNase A (100 µg/mL) and PI (5 µg/mL) in the dark for 30 min at room temperature. Samples were analysed by flow cytometry (FACSCalibur, Becton Dickinson). The green fluorescence FL1-H and red fluorescence FL2-A of 5 × 10^3^ events per sample were measured with the use of a laser beam at 530 ± 30 nm (λex = 488 nm) or 585 ± 21 nm (λex = 488 nm), respectively.

### 4.7. Caspase-3 and Caspase-8 Activity Assays

The activity of caspase-3 and caspase-8 was measured by flow cytometry using FLICA assay commercial kits which contained fluorochrome-labelled inhibitors of caspases. The cells (10^5^/mL) were incubated at 37 °C for 72 h in the presence of MNP or MDION used at IC_50_ or IC_90_, respectively. Thereafter, they were stained using the selective inhibitor of the active form of caspase-3 (Asp-Glu-Val-Asp-fluoromethyl ketone, DEVD-FMK) or caspase-8 (Ile-Glu(OMe)-Thr-Asp(OMe)-fluoromethyl ketone, IETD-FMK), respectively, conjugated with fluorescein isothiocyanate (FITC) for 30 min at 37 °C and analysed by flow cytometry (FACScan; Becton Dickinson). The green fluorescence (FL-1) of 5 × 10^3^ events per sample was measured with the use of a laser beam at 530 ± 15 nm (λ_ex_ = 488 nm). The data were analysed using the BD CellQuest Pro (Becton Dickinson) as well as FSC Express, version 4.0 (DeNovo) software. In addition, control assays were carried out at 72 h to confirm 50% and 90% inhibition of cell growth by the studied compounds, respectively, by counting the viable cells in the presence of trypan blue with the use of a Burker hemocytometer.

### 4.8. Lysosomal Integrity Assay

Lysosomal integrity assays were performed with the use of the lysosomotropic probe LysoTracker Green DND-26. The cells (10^5^/mL) were incubated at 37 °C in the presence of MNP or MDION used at IC_20_ or IC_50_, respectively, and at the indicated time points (0, 1, 4, 8, 12, 24, 48, and 72 h) they were stained for 30 min with 100 nM LysoTracker Green DND-26. Subsequently, the samples were analysed by flow cytometry (FACScan; Becton Dickinson). The green fluorescence (FL-1) of 5 × 10^3^ events per sample was measured with the use of a laser beam at 530 ± 15 nm (λ_ex_ = 488 nm). The data were analysed using the BD CellQuest Pro (Becton Dickinson) as well as FSC Express, version 4.0 (DeNovo) software. In addition, control assays were carried out at 72 h to confirm 20% and 50% inhibition of cell growth by the studied compounds, respectively, by counting the viable cells in the presence of trypan blue with the use of a Burker hemocytometer.

### 4.9. Statistical Analysis

The normality of the data distribution was verified using the Shapiro–Wilk test. Results are presented as the mean ± SD for data having the normal distribution or median (interquartile range) for non-normal data of at least three independent experiments. Statistical analysis of the significance level of differences observed between analysed values was carried out using the parametric Student’s *t*-test or the non-parametric Mann–Whitney *U* test, respectively. A *p*-value less than 0.05 was considered a significant difference.

## 5. Conclusions

The findings of the study showed that the pyridinium salts, MNP and MDION, exerted very similar antitumour activities against sensitive leukaemia HL60 cells and their multidrug resistant topoisomerase II-defective HL60/MX2 counterparts. They modulated the cellular ROS level and significantly increased the percentage of the sub-G1 subpopulation of sensitive HL60 and resistant HL60/MX2 cells. However, the induction of DSB formation was not a significant mechanism of action of MNP and MDION in sensitive HL60 and resistant HL60/MX2 cells. Both studied compounds triggered apoptosis of sensitive HL60 cells and their MDR counterparts with the participation of caspase-3 and caspase-8. Additionally, the results found for MDION, which is highly active in stimulating ROS production, indicated that lysosomes may also be involved in the programmed death of sensitive HL60 and resistant HL60/MX2 cells induced by this agent. However, because multiple molecular players can be involved in cellular events triggered by MNP and MDION, further in-depth systematic studies are required to dissect cell death pathways activated by these pyridinium salts. In addition, in order to draw some general conclusions explaining the impact of structural factors on the activity of pyridinium salts, including MNP and MDION, towards sensitive HL60 and resistant HL60/MX2 cells, a larger series of these salts should be obtained to perform a “structure-activity relationship” (SAR) analysis. Moreover, further studies using other haematological cancer cell lines and solid tumour cell lines, as well as their sublines resistant to conventional drugs, are needed to assess the therapeutic potential of MNP and MDION in the cure of oncological patients.

## Figures and Tables

**Table 1:** The ability of MNP and MDION to inhibit the growth of sensitive HL60 and resistant HL60/MX2 leukaemia cells.

Compound	HL60 Cells			HL60/MX2 Cells			
	IC_20_ {µM}	IC_50_ {µM}	IC_90_ {µM}	IC_20_ {µM}	IC_50_ {µM}	RF	IC_90_ {µM}
MNP	7.5 ± 0.9	24.3 ± 7.2	82.1 ± 25.3	7.2 ± 1.4	20.5 ± 5.2	0.84	77.3 ± 12.8
MDION	15.0 ± 1.9	80.5 ± 10.5	573.0 ± 171.4	17.0 ± 3.4	95.5 ± 19.9	1.19	591.2 ± 164.0

IC_20_, IC_50_, and IC_90_ values are the MNP or MDION concentrations required to inhibit 20%, 50%, and 90% of cell growth, respectively. Resistance factor (RF) was calculated as RF = IC_50_(R)/IC_50_(S) (S—sensitive HL60 cells; R—resistant HL60/MX2 cells). The values represent the mean ± SD of five independent experiments (made in triplicate).

## Data Availability

Not applicable.

## Sample Availability

Not available.

## Abbreviations

2′,7′-DCF-DA—2′,7′-dichlorofluorescein diacetate; DOX—doxorubicin; DSB—double strand break; FITC—fluorescein isothiocyanate; IC—inhibitory concentration; IDA—idarubicin; LMP—lysosomal membrane permeabilization; MDION—3,3,6,6,10-pentamethyl-3,4,6,7-tetrahydro-[1,8(*2H,5H*)-dion]acridine chloride; MDR—multidrug resistance; MNP—1-methyl-3-nitropyridine chloride; MRP1—multidrug resistance-associated protein 1; MX—mitoxantrone; P-gp—P-glycoprotein; PI—propidium iodide; RF—resistance factor; ROS—reactive oxygen species; VINC—vincristine.
